# Impact of the Respiratory Microbiome on Host Responses to Respiratory Viral Infection

**DOI:** 10.3390/vaccines5040040

**Published:** 2017-11-03

**Authors:** Maxime Pichon, Bruno Lina, Laurence Josset

**Affiliations:** 1 Hospices Civils de Lyon, Centre National de Reference des virus des Infections Respiratoires France Sud, Laboratoire de Virologie, Institut des Agents Infectieux, Groupement Hospitalier Nord, F-69317 Lyon CEDEX 04, France; maxime.pichon01@chu-lyon.fr (M.P.); bruno.lina@chu-lyon.fr (B.L.); 2 Université de Lyon, Faculté de Médecine Lyon Est, Centre International de Recherche en Infectiologie (CIRI), Inserm U1111, Centre National de la Recherche Scientifique (CNRS) UMR5308, Ecole Nationale Supérieure de Lyon (ENS), équipe Virpath, F-69372 Lyon CEDEX 08, France

**Keywords:** respiratory tract, viral infections, respiratory microbiome, 16S, whole genome sequencing, NGS

## Abstract

Viruses are responsible for most of both upper and lower acute respiratory infections (ARIs). The microbiome—the ecological community of microorganisms sharing the body space, which has gained considerable interest over the last decade—is modified in health and disease states. Even if most of these disturbances have been previously described in relation to chronic disorders of the gastrointestinal microbiome, after a short reminder of microbiome characteristics and methods of characterization, this review will describe the impact of the microbiome (mainly respiratory) on host responses to viral ARIs. The microbiome has a direct environmental impact on the host cells but also an indirect impact on the immune system, by enhancing innate or adaptive immune responses. In microbial infections, especially in viral infections, these dramatic modifications could lead to a dramatic impact responsible for severe clinical outcomes. Studies focusing on the microbiome associated with transcriptomic analyses of the host response and deep characterization of the pathogen would lead to a better understanding of viral pathogenesis and open avenues for biomarker development and innovative therapeutics.

## 1. Introduction

### 1.1. Burden of Acute Respiratory Infections (ARI)

Acute respiratory infections (ARIs) are the most common type of acute infection worldwide in both adults and children and are mainly caused by viruses, including respiratory syncytial virus (RSV), human rhinovirus (hRV), influenza virus (IV), or metapneumovirus (hMPV). In Europe, lower respiratory tract infection is the eighth leading cause of disease in both adults and children [1]. ARIs account for an estimated 40% of all healthcare-associated infections in pediatric long-term care facilities [2]. Most of these viral infections could lead to very severe forms of respiratory diseases. For example, it is estimated that 33.8 million new episodes of RSV-associated acute respiratory infection occurred worldwide in children younger than 5 years of age, during the 2005 epidemics, with at least 10% of these children necessitating hospital admission [3].

Attempts to prevent respiratory viral infections are currently limited to vulnerable high-risk groups such as immunocompromised patients, the elderly, or young children and include optimized vaccination, reduced exposure and prophylactic treatment. Because of the burden, the clinical presentation and the difficulties with anticipating clinical complications of respiratory infections, it is necessary to better understand their pathophysiology.

### 1.2. The Respiratory Microbiome: Still a Lot of Unknowns

The human microbiota comprises all microorganisms (bacteria, viruses, fungi, *archae*) that exist in the human body. Despite numerous studies on the microbiome, the definition of this term is still controversial. From a genomic perspective, the microbiome can be considered the collection of the microbiota genomes [4]. From an ecological perspective, the microbiome corresponds to the whole ecological niche considered (-biome), including both the microbiota and the habitat it colonizes. While most of the studies on the microbiome have only described its bacterial component, the human body is colonized by more viruses than bacteria and also comprises fungi and *archeae* [5,6]. Given that no study currently describes the composition and the role of the respiratory virome and mycobiome during ARI, we will focus on the bacterial microbiota only in this review.

Extensive studies in the general population have focused on the characterization of the bacterial microbiota per niche. These studies have obtained important information on the structure and variation of the composition over time of the different microbiomes. It is important to acknowledge that the baseline microbiome varies among normal individuals and that colonization by certain bacteria appears to be conferring either a protective or deleterious effect on subsequent viral infection (described in Table 1). However, contrary to the gastrointestinal tract or skin niches, it is only recently that the respiratory tract microbiome has been studied.

**Figure 1:**
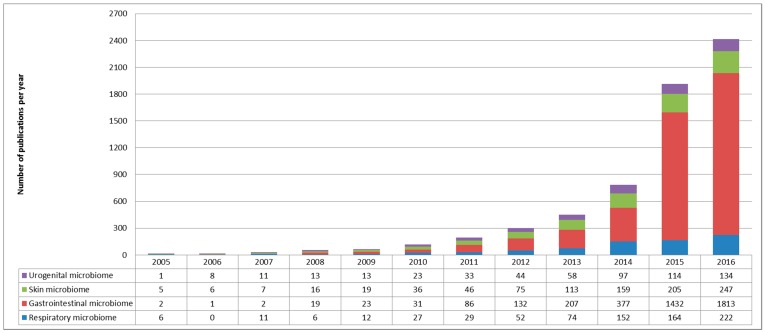
Bibliometric parameters of microbiome studies. Representations were limited to the four most abundant microbiomes (urogenital tract in purple, skin microbiome in green, gastrointestinal tract in red and respiratory microbiome in blue). The number of publication focusing on microbiome increased slightly until 2011, after which Next Generation Sequencing (NGS) technologies became more widely available. Microbiome publications focused mainly on gastrointestinal microbiome; the other three niches being studied in the same proportions.

Although more than 7000 articles were associated with the “microbiome” keyword in PubMed, with a slight part focusing on respiratory tract microbiome, only a small proportion focused on microbiome disturbance in viral infections, especially in respiratory tract infections (Figure 1).

## 2. Impact of the Respiratory Microbiota on Viral Infection Pathogenesis

After a first period focusing on the characterization of the composition and the physiological variations of the “healthy” respiratory microbiome, most recent studies have focused on changes induced during a so-called “pathological” situation, infectious or not. Unfortunately, only few studies have compared baseline healthy pre-infection microbiome and post-infection modified microbiome in acute infections as it is very difficult to get human samples before viral infection, except in challenge studies [7,8]. Animal studies provide some insight into the mechanisms, yet their respiratory microbiome is very different to the human microbiome and in addition, while there are lots of studies describing bacteria-virus interactions between defined pathogens, only a few studies have analyzed trans-kingdom interactions within the respiratory microbiota. For example, it has been well established that pneumococcus super infection following IV infection dramatically increases the lethality of influenza in a mouse model and in humans [9,10]. However, how the whole respiratory microbiota is associated with influenza severity is still largely unexplored [11,12,13]. The respiratory microbiota could impact ARI outcome through bacteriome-bacteriome or bacteriome-virome interactions (Table 1). Interactions between bacteriophages and the bacteriome are increasingly being described in the GI. Bacteriophages could alter the bacteriome composition and function through both lytic and lysogenic infections of their hosts [14]. In addition, bacteriophages binding to the intestinal mucosae might provide some form of acquired immunity to invading pathogens [15]. However, there are currently no data available describing interactions between the prokaryotic virome and the bacterial microbiota in the respiratory tract. Therefore, this review describes only interactions between bacterial microbiota and a single viral pathogen.

### 2.1. Bacterial Microbiota Direct Impact on the Respiratory Tract Environment

Several studies indicate that the lung bacterial microbiota influences lung morphology, function and mucosal lymphoid tissue development [16,17]. During ARI, the respiratory bacterial microbiota may also directly impact on the respiratory tract environment to influence viral pathogenesis. Using bacterial whole genome sequencing, Leung et al. have studied the respiratory microbiome of patients infected by IV compared to that of uninfected patients with the same demographic characteristics [18]. These metatranscriptomic data have shown that IV-infected patients have a modified bacterial microbiome. These bacteria contain more mobility genes, flagella-assembling genes and/or response-to-chimiotactism genes compared to the same bacteria in non-infected patients. The existence of a selection process for metabolically-adapted bacteria could be evoked and may lead to bacteria-induced mucocilliar alterations and secretion of immunomodulating metabolites limiting the host response. The presence of these species was associated with severe anatomical consequences, leading to very poor clinical outcomes for the infected hosts (respiratory distress or even death).

Bacterial microbiota may also promote viral infection and/or pathogenesis by a direct effect on epithelial cells. For instance, *Haemophilus influenzae* infection can lead to a severe increased expression of ICAM-1 and Toll-like receptors 3 (TLR-3) by the pulmonary epithelial cells. TLR3, as other TLR, is implicated in viral genome detection and immunity and so is responsible for host response to infection via immunological pathways such as the NF-κB pathway. In addition, overexpression of the main receptor of hRV, ICAM-1, has been associated with enhanced hRV binding and RV-induced chemokine responses, leading to an increased pathogenesis [26].

Finally, bacterial microbiota could also interact directly with viral pathogens. Such an interaction has not been described in the respiratory tract but it is known that some enteric viruses bind bacterial lipopolysaccharides (LPS) to stabilize their entry into the target cells or to induce unresponsiveness to itself [27,28].

### 2.2. Impact on Innate and Adaptive Immune Responses to Infection

Most symptoms occurring during acute respiratory infection are due to the excessive intensity of the host inflammatory response. Nasal and systemic levels of several pro-inflammatory cytokines have been associated with clinical outcome in influenza for example [29,30]. Increasing evidence suggest that the respiratory microbiome interact with the immune system and therefore modulate clinical outcomes of ARI.

In animal models, Wang et al. reported the impact of the respiratory microbiome on the host resistance to influenza virus [11]. Increased morbidity and lethality was observed in specific-pathogen-free (SPF) mice compared with wild-type mice after infection with IV. Wild-type mice resistance to influenza was shown to be provided by continual TLR-2 stimulation by *Staphylococcus aureus,* stably colonizing the respiratory tract. This in turn would allow lung recruitment of M2 alveolar macrophages inhibiting influenza-mediated lethal inflammation In addition, using another mouse model, Wu et al. have found that oral administrations of probiotic treatment significantly restored the immune response and enhanced the activation of numerous pathways, mainly TLR-7 and NF-κB, implicated in single stranded RNA virus recognition [31]. This study demonstrates that modification of the gastrointestinal tract microbiome has an important impact on respiratory tract infections. Even if this modification has not been clearly identified in respiratory infections, these promising data suggested that microbiota modification could lead to development of innovative therapies of respiratory infections. Taken together, data obtained from animal models suggest that influenza pathogenesis on a respiratory tract with modified microbiome is associated with a non-efficient local immune response. Conversely, an unmodified microbiome allows the maintenance of effective innate response needed for the development of an appropriate adaptive response against IV. Microorganism viability does not seem to be necessary as in a mouse model it has been established that LPS administration could be sufficient to restore an adaptive immune response in an altered host [12]. The hypothesis is that the microbiome can trigger many Pattern Recognition Receptors and stimulate leukocytes at the local and systemic level using isolated proteins acting as antigens. In an SPF mouse model, colonization of the upper respiratory tract by commensal bacteria significantly reduced the lung alterations associated with influenza virus infection. This colonization, by decreasing the lethality of influenza infection via modification of the monocyte response phenotype, induces a less destructive macrophage activation phenotype [11]. 

In another study, Abt et al. have found that wild-type mice pretreated with an antibiotic cocktail, denaturing their “natural” microbiome, exhibited increased morbidity during infection with the A(H1N1)pdm09 virus (greater weight loss and significant desaturation) [32]. The severity of these diseases is associated with two main factors: reduction of the dendritic cell migration rate and reduction in the number of local T-cell lymphocytes. When ineffective, these factors, essential for a functional immune response, contributed to maintain a high viral titer.

In a prospective cohort study of the nasopharyngeal microbiota associated with RSV severity in children less than 2 years of age hospitalized for bronchiolitis, nasopharyngeal bacterial microbiota dominated by *Haemophilus* and *Streptococcus* have been reported to be associated with an exaggerated systemic response and with increase RSV severity. This exacerbation was associated with overexpression of TLR gene pathways and genes implicated in neutrophil response during RSV infection [21]. In this study, infants with NP microbiota dominated by *Staphylococcus aureus* showed a limited immune response and experienced mild RSV infection. The level of interleukin (especially IL-6, -8 and -17A) was correlated with the bacterial species dominating the NP microbiota. However, this study could only show association and not causation, between NP microbiota and RSV severity. Further studies are needed to decipher whether NP microbiota changes are responsible for increased immune response and RSV severity. Some insights about causation were provided by a large prospective longitudinal study following the NP microbiota of infants at 2, 6 and 12 months and during ARI [22]. The authors were able to show that early NP colonization with *Moraxella* or *Streptococcus* was associated with an earlier first episode of upper or lower respiratory tract infection, respectively.

In addition, there have been numerous studies describing the association between *Moraxella* respiratory tract colonization and increased severity of RSV infection, with RSV being reciprocally able to strongly alter the respiratory microbiome of the host [22,23,24,33]. During winter, co-infections combining RSV and *Moraxella* are not uncommon because these two pathogens co-circulate during the coldest seasons. Such co-infections are described as being associated with more frequent otitis media and it is suggested that viral infection increases the incidence of bacterial super infection.

**Figure 2:**
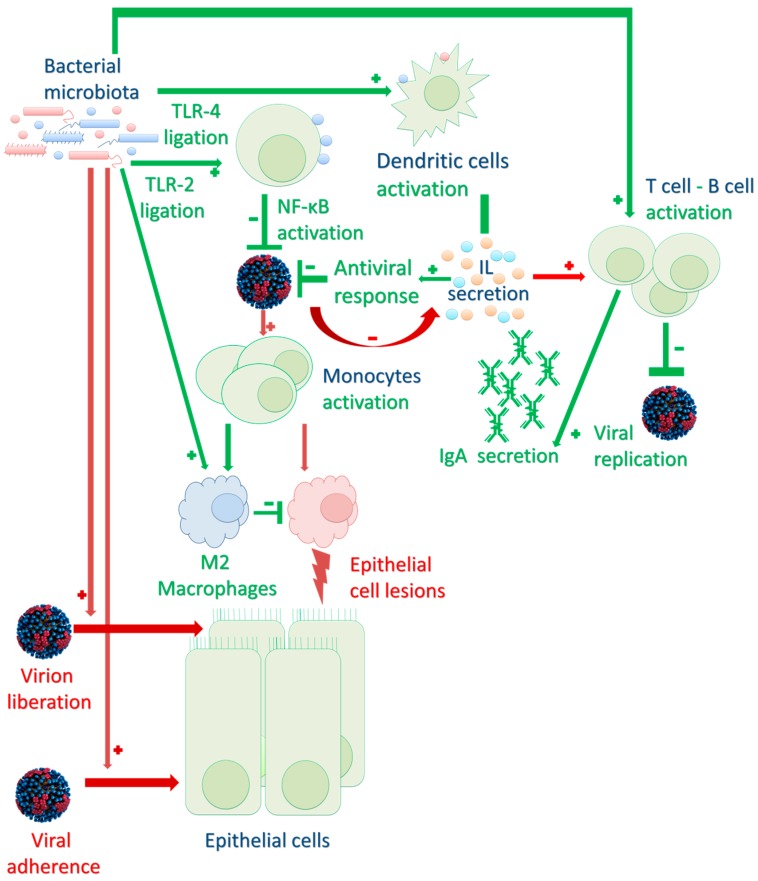
Schematic representations of the host immune system and respiratory microbiome during respiratory viral infections. Red and green arrows and symbol symbolize interactions responsible for severe or mild diseases respectively. Bacterial microbiota could have a direct effect on viral infection (enhancing virion liberation or virion adherence to a new host cell), or an indirect effect on immune response to viral response to infection (activating Toll-like receptors TLR, leading to cellular activation of dendritic cells B/T lymphocytes and monocytes) via interleukin (IL) secretion. Bacterial microbiota has an impact on both innate (inflammation limited by a less-destructive M2 phenotype of macrophages) or adaptive (immunoglobulin A – IgA -, secretion). Viral adhesion enhancement by bacterial microbiota has been well-established in gastro-intestinal infections but need to be studied especially in respiratory infections.

For the time being, published studies have only partially explored the complex mechanisms associating the microbiome with the immune response to ARI. Only limited interactions between viral pathogens and commensal bacteria have been described (summarized in Figure 2). According to some authors, these interactions and the description of the respiratory microbiome could be used in clinical practice to optimize management of patients, especially when severe consequences could occur.

## 3. Clinical Use of the Respiratory Microbiome Impact during Viral Infections

### 3.1. Respiratory Microbiome Characterization: Usable as a Prognostic Biomarker?

Microbiome-associated biomarkers have been suggested to predict respiratory disease severity even before the description of the microbiome concept. For years, it has been observed in patients with cystic fibrosis that detection and an increase in the proportion of some bacterial species, such as *Pseudomonas aeruginosa* in the respiratory tract marked a negative turning point in the evolution of their disease [34].

Similarly, and even if data are still limited, it has recently been reported in children that nasal carriage of *Streptococcus pneumoniae* was positively associated with seroconversion to hMPV, without any association with lethality or morbidity [20]. In addition, in a large cohort of RSV-infected neonates, Teo et al. found that the microbiome could be used for the early detection of both ARI symptoms and chronic wheezing that are consequences of repetitive or severe bronchiolitis [22]. Interestingly, early NP colonization (before 2 months of age) with *Streptococcus* was associated with increased risk of wheezing at 5 years old. This discovery could lead to the possible use of microbiota characterization as a biomarker of chronic wheezing, many years after the initial infection.

In a recently published study, we characterized the nasopharyngeal bacterial microbiota associated with severe influenza in children. Patients visiting the emergency room for acute influenza (symptom onset ≤ 2 days) were sampled at hospital arrival and we were able to define a microbial signature that distinguished those patients that had developed severe and mild influenza. This signature included *Staphylococcus aureus*, *Prevotella* spp., *Streptobacillus* spp., *Porphyromonas* spp., *Granulicatella* spp., *Veillonella* spp., *Fusobacterium* spp., *Lachnospiracea incertae sedis* and *Haemophilus* spp. [13]. With the exception of *Staphylococcus aureus*, all these bacteria were related to a severe form of the disease. Moreover, in severe forms, *Moraxella catarrhalis* and *Parvimonas micra* seemed more associated with respiratory complications, whereas *Actinomyces* spp., *Corynebacterium* spp., *Dolosigranulum pigrum*, *Chryseobacterium* spp. and *Prevotella* spp. were more associated with the neurological form of the disease. The modification of the satellite microbiome using adapted therapies to restore a less severe microbial signature could lead to a modification of the clinical outcome. Furthermore, such a microbial signature could also be used at the time of influenza diagnosis to improve the identification of patients at risk of severe disease and to allow the development of risk-based strategies for enhanced care management. A French multicenter study launched in 2017 aims to confirm the presence of this microbial signature and its applicability in clinical practice in pediatric cohorts. Doing so, we would prove the benefit of using the microbiome as a prognostic marker of severe forms of viral respiratory diseases.

No causal relation between the nasopharyngeal microbiome and the immune response to viral infections could be deduced from the studies described in this subsection. However, these data suggest that microbiome characterization could be useful for predicting clinical outcomes. We could therefore imagine that therapeutic modification of the microbiome, by treatment administration or vaccination, could be of interest in severe infectious disease management. This approach is very promising, especially in viral infection management, where treatments are very limited [35,36].

### 3.2. Respiratory Microbiome: A Therapeutic Target?

Several studies suggest that the respiratory microbiome may be a promising target for ARI prophylaxis and treatment. Some authors suggest that bacterial antigens could be used as a therapeutic approach against IV infections [12]. In a mouse model, it has been shown that animals, pretreated with aerosols of bacterial lysates (containing LPS), exhibited faster and more effective inflammatory responses after influenza virus infection. The LPS-induced response reduces tissue damage associated with viral infection and thereby improves the survival of infected animals [37]. However, these data are still debated; while it is well-known that excessive inflammation following influenza virus infection is responsible for the most severe disease (cytokine storm process) immunosuppressive treatments have been found to be ineffective against influenza in clinical trials [38]. Similarly to sepsis, the timing of immunomodulatory intervention is likely to be critical for successful influenza management [39].

Similarly, activation of TLR-2, -6 and -9 by synthetic agonists (nasally administered) reduce *Parainfluenza* virus titers. This activation is not associated with reduction of the symptomatology due to bronchial hyper reactivity [40]. Similarly, the administration of inactivated toxins of *Escherichia coli* has been reported to be effective for protection against RSV and IV and the administration of *Lactobacillus rhamnosus,* prior to an RSV challenge, is reported to limit pulmonary destruction due to the virus [41,42]. These *Lactobacilli* were be useful to protect against excessive inflammation during infection by altering both the production of many mediators (interferons and interleukins) and activation of dendritic cells via expression of CD103 and CD11b [43]. Conversely, an indirect antiviral effect of antibiotic administration (erythromycin) during rhinovirus infections could be explained by a disturbance of bacteria-virus interactions. Reduction of the expression of ICAM by the epithelial cells induced by the antibiotic, limit the binding of rhinovirus. Moreover, the decrease of TLR-3 expression, responsible for most of the rhinovirus-induce damages, limit the viral-induced lung destruction [44].

Although these studies have demonstrated very promising results, there is still a long way to go before using respiratory microbiome modification or enrichment to prevent or treat respiratory viral infections. To develop these therapies, future studies have to be carried out on large cohorts before the routine use of these technologies.

## 4. Conclusions

The development of Next Generation Sequencing (NGS) technologies has led to a dramatic increase in microbiome understanding in physiological and pathological situations. Unlike the previous approach to infectious diseases, which focused on the pathogen, this approach takes into account all the actors (target cells response, immune system activation, therapeutic molecules administration…) and human body organization as a niche. Because most diseases are caused by an imbalance of these actors, it becomes necessary to standardize studies of any ecological niches. Using a global approach, especially in viral diseases studies, provides more valid data, even though no consensual approach is currently accepted in the scientific community as a whole.

**Figure 3:**
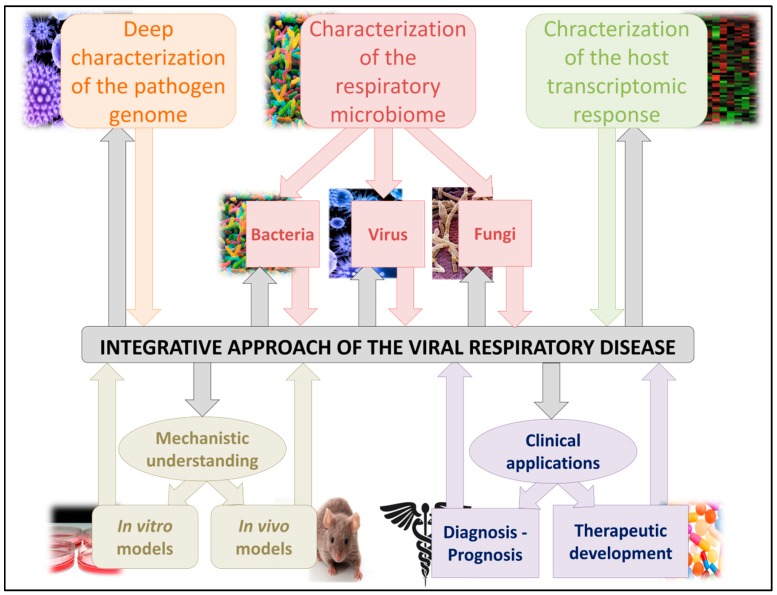
Integrative scheme of studies focusing on respiratory virus infections. All three actors of the viral disease need to first be completely characterized (microbiome characterization for the environment, viral genome to study the pathogen itself and transcriptomic analyses for the host response). These interactions between host, its microbiome and the pathogen are very important in viral infections pathogeneses. After a complete characterization of these parameters, mechanistic understanding, using animal models, is needed before using these data in clinical diagnosis optimization or for the development of new therapies.

From the earliest paradigms of infectious diseases studies, it has been noteworthy that optimization in the clinical management of infected patients will need to best characterize the pathogen involved. The development of high-throughput NGS technologies makes it possible to finely study the pathogen genome, be this in terms of consensus or minority variant sequence analyses. As we have demonstrated in this review, the microbiome, whether respiratory or not, could be strongly implicated in viral pathogenicity and could be finely studied, using these technologies, to understand its implications. Furthermore, due to the implication of host immunity in the protection against but also in the development of infectious viral disease, the study of the cellular response to the transcriptomic infection is not to be neglected. This integrative approach is essential for the overall understanding of viral infections, particularly in respiratory diseases and is described in Figure 3. Only after consolidation of the mechanistic understanding, using animal experimentation, will it be possible to consider microbiome applications to clinical pathologies management. The understanding of the microbiome will open a new field of clinical management and therapeutic approaches, with the development of new diagnosis tests or therapeutic molecules.

## Figures and Tables

**Table 1:** Microbiome-specific bacteria implicated in or modified during viral infections. IV: Influenza virus; MPV: Metapneumovirus; RV: Rhinovirus; RSV: Respiratory Syncytial Virus. The main viruses responsible for respiratory infections (IV, RSV and RV) are also the most studied. It is interesting to note that the increase in considered bacteria exists independently of the observed virus (*Lactobacillus rhamnosus*, *Escherichia coli* and *Staphylococcus aureus* are protective whatever the viral model; the presence of *Streptococcus pneumoniae* is associated with an increase of the complication rate or seroconversion). Outcome “complication”: disease worsening; outcome “Protection”: indicate a limitation of the disease complications.

Virus	Model	Sample Site	Bacteria	Consequences	Outcome	Reference
**IV**	*Human clinical samples*	Induced sputum	*Acinetobacter baumanii*	Bacteria species increasing during A(H1N1) infection	No modification	Leung et al. [18]
			*Bacillus* spp.			
			*Pseudomonas aeruginosa*			
	*Human clinical samples*	Induced sputum	*Neisseria* spp.	Bacteria species decreasing during A(H1N1) infection	No modification	Leung et al. [18]
			*Prevotella* spp.			
	*Mouse model*	Lung homogenates	*Streptococcus pneumoniae*	Increased severity	Complication	McCullers et al. [10]
	*Human clinical samples*	Nasopharyngeal samples	*Streptobacillus* spp.	Predict a severe form of influenza in children	Complication	Langevin et al. [13]
			*Porphyromonas* spp.			
			*Granulicatella* spp.			
			*Fusobacterium* spp.			
			*Lachnospiracea* spp.			
			*Haemophilus* spp.			
	*Human clinical samples*	Nasopharyngeal samples	*Staphylococcus aureus*	Predict a benign form of influenza in children	Protection	Langevin et al. [13]
	*Human clinical samples*	Nasal swabs	*Lactobacillus helveticus*	Correlated with higher H1 IgA antibody response	Protection	Salk et al. [19]
			*Bacteroides ovatus*			
	*Human clinical samples*	Nasopharyngeal samples Nasal swabs	*Veillonella dispar*	Predict a severe form of influenza in children Correlated with lower H1 IgA antibody response	Protection	Langevin et al. [13] Salk et al. [19]
	*Human clinical samples*	Nasopharyngeal samples Nasal swabs	*Prevotella melaninogenica*	Predict a severe form of influenza in children Correlated with Higher H1 IgA antibody response	Complication—Protection	Langevin et al. [13] Salk et al. [19]
	*Human clinical samples*	Nasal swabs	*Streptococcus infantis*	Correlated with Higher H1 IgA antibody response	Complication	Salk et al. [19]
**MPV**	*Cellular model*	Cellular model	*Streptococcus pneumoniae*	Increased seroconversion rate during challenge	Protection	Verkaik et al. [20]
**RSV**	*Human clinical samples*	Nasopharyngeal samples	*Haemophilus influenzae* *Streptococcus* spp.	Increased hospitalization rate	Complication	De Steenhuijsen Piters et al. [21]
	*Mice model—Human clinical samples*	Nasopharyngeal samples	*Moraxella catarrhalis*	Increased severity	Complication	Teo et al. [22] Beura et al. [23] Furusawa et al. [24]
	*Mice model* *Human clinical samples*	Nasopharyngeal samples Lung homogenates	*Staphylococcus aureus*	Decreased complications	Protection	De Steenhuijsen Piters et al. [21] Wang et al. [11]
	*Cellular model*	Cellular model	*Escherichia coli*	Increased protection against infection	Protection	Cagno et al. [25]
**RV**	*Cellular model*	Cellular model	*Haemophilus influenzae*	Entry point production via ICAM-1 and TLR-3 production	Complication	Sajjan et al. [26]
